# Detection of Low-Density Foreign Objects in Infant Snacks Using a Continuous-Wave Sub-Terahertz Imaging System for Industrial Applications

**DOI:** 10.3390/s24227374

**Published:** 2024-11-19

**Authors:** Byeong-Hyeon Na, Dae-Ho Lee, Jaein Choe, Young-Duk Kim, Mi-Kyung Park

**Affiliations:** 1 School of Food Science and Biotechnology, Kyungpook National University, Daegu 41566, Republic of Korea; hyeonbest@knu.ac.kr (B.-H.N.); dleogh3046@knu.ac.kr (D.-H.L.); jane100497@naver.com (J.C.); 2 ICT Research Institute, Daegu Gyeongbuk Institute of Science & Technology (DGIST), Daegu 42988, Republic of Korea; ydkim@dgist.ac.kr

**Keywords:** sub-terahertz imaging, low-density foreign objects, on-site detection, infant snacks

## Abstract

Low-density foreign objects (LDFOs) in foods pose significant safety risks to consumers. Existing detection methods, such as metal and X-ray detectors, have limitations in identifying low-density and nonmetallic contaminants. To address these challenges, our research group constructed and optimized a continuous-wave sub-terahertz (THz) imaging system for the real-time, on-site detection of LDFOs in infant snacks. The system was optimized by adjusting the attenuation value from 0 to 9 dB and image processing parameters [White (W), Black (B), and Gamma (G)] from 0 to 100. Its detectability was evaluated across eight LDFOs underneath snacks with scanning at 30 cm/s. The optimal settings for puffed snacks and freeze-dried chips were found to be 3 dB attenuation with W, B, and G values of 100, 50, and 80, respectively, while others required 0 dB attenuation with W, B, and G set to 100, 0, and 100, respectively. Additionally, the moisture content of infant snacks was measured using a modified AOAC-based drying method at 105 °C, ensuring the removal of all free moisture. Using these optimized settings, the system successfully detected a housefly and a cockroach underneath puffed snacks and freeze-dried chips. It also detected LDFOs as small as 3 mm in size in a single layer of snacks, including polyurethane, polyvinyl chloride, ethylene–propylene–diene–monomer, and silicone, while in two layers of infant snacks, they were detected up to 7.5 mm. The constructed system can rapidly and effectively detect LDFOs in foods, offering a promising approach to enhance safety in the food industry.

## 1. Introduction

During manufacturing or packaging processes, food products can become contaminated with foreign objects such as insects, metals, plastics, rubber, sand, hair, glass, nails, and any other extraneous substances [1]. Foreign objects are materials that are unintentionally introduced into food that need to be controlled and prevented. Foreign object contamination leads to conflicts and quality issues between manufacturers and consumers, as well as illness or injury, resulting in persistent and numerous recalls [2]. The Consumer Complaint Monitoring System (CCMS) reported that these foreign objects are the biggest source of consumer complaints, comprising 45% of all complaints in 2017 [3]. Ingesting foreign objects can lead to vomiting, discomfort when swallowing, pneumonia, or more severe complications such as bronchiectasis, hemorrhage, and peritonitis. Especially, infants are one of the most fatal and problematic groups when exposed to low-density foreign objects (LDFO) due to a lack of posterior dentition and poor chewing abilities [4,5].

In the food industry, a foreign object inspection in the packaging process is typically performed using a metal detector and X-ray system due to their advantages of user-friendliness and cost-effectiveness [6]. However, metal detectors are limited to detecting only metallic objects, while X-ray systems [2], though able to detect dense foreign objects, carry inherent risks due to ionizing radiation exposure for both operators and food products [7,8,9]. To address the limitations of these traditional detection methods, non-ionizing technologies, such as microwave (MW), and millimeter-wave (mm-wave) imaging have been developed for detecting non-metallic foreign objects [8,9,10,11]. However, MW and mm-wave offer relatively lower spatial resolution due to their lower frequencies of 0.3 GHz to 300 GHz and 30 GHz to 300 GHz, respectively, making it challenging to detect fine structures and small LDFOs such as plastic, insect, and rubber [12]. Thus, a new inspection method is needed for the detection of LDFO in the food industry.

Since the emergence of THz technology in the early 1990s, it has been advanced and extended to detect LDFOs, competing with X-ray detectors [13]. The THz region of the electromagnetic spectrum is located between the microwave and infrared (IR) regions, with a frequency range of 0.1 to 10 divided into sub-THz waves (0.1–0.3 THz) and a THz wave (0.1–10 THz) [14]. The sub-THz waves can penetrate various objects, and the unique fingerprint image of each foreign object becomes visible owing to intermolecular and intramolecular rotation and vibrational transitions at sub-THz frequencies [15,16]. Thus, sub-THz waves are an excellent nonionizing alternative to X-rays because of their ability to generate high-resolution images of nonmetallic objects and LDFOs. More importantly, their nonionizing properties guarantee safety for humans [17,18]. There are two primary methods for detecting foreign objects using sub-THz waves: continuous-wave (CW) sub-THz which uses a single-frequency wave, and THz time-domain spectroscopy (TDS), which utilizes broadband pulsed waves [19]. Although TDS systems offer enhanced analytical detectability, including the ability to perform depth profiling by analyzing the time delay in reflected signals, complexity and cost limit their practicality for certain industrial applications [20]. Thus, CW sub-THz has found widespread application in the detection of foreign objects in food because of the simplicity and fast detection system [21].

For the application of CW sub-THz waves, a CW sub-THz imaging system installed with a Gaussian beam source and a raster scanner was developed for detecting maggots and crickets embedded in noodle powder [22]. Another group also developed a CW THz imaging system equipped with the same Gaussian beam and raster scanner for the successful detection of maggots and crickets embedded in milk powder [23]. In a subsequent study [21], the same research group improved the previous version of the CW sub-THz imaging system by utilizing a Bessel-Gauss beam for the successful identification of polystyrene, rubber bands, pepper seeds, and metal embedded in chocolate and black seaweed within 6 min by extending the scan area. The non-diffractive nature of the Bessel–Gauss beam allowed the imaging system to maintain focus over a larger area, enabling consistent imaging without frequent adjustments. However, despite the successful detection of various LDFOs using CW sub-THz imaging systems with faster and high-resolution images, the application of CW sub-THz imaging detection in the food industry is still limited in its practical application owing to the limitations of resolution and acquisition speed for foreign objects [24].

The purpose of this study is to construct a CW sub-THz system and demonstrate its applicability to the rapid, on-site detection of LDFOs in infant snacks by practical application. To achieve this goal, a cost-effective, on-site applicable CW sub-THz imaging system was developed, incorporating a linear scanner, cylindrical concave mirror, sub-THz source, conical horn antenna, and movable conveyor belt operating at 30 cm/s. This system was employed to detect various LDFOs, including ethylene–propylene–diene monomer (EPDM), polyurethane (PU), polyvinyl chloride (PVC), silicone, houseflies, cockroaches, and *Penicillium citrinum* in infant snacks, demonstrating its practical usability in food industry applications. Therefore, the main contributions of this paper are as follows:

1. The potential of sub-THz imaging as a non-ionizing alternative for detecting LDFOs is discussed.
2. A CW sub-THz imaging system is constructed for the detection of LDFOs in infant snacks.
3. The system’s ability to detect various LDFOs in infant snacks is demonstrated, and its real-time detection performance and feasibility for industrial applications are evaluated.

The rest of the paper is organized as follows. Section 2 describes the experimental setup and methodology including the construction of the CW sub-THz imaging system. Section 3 presents the results of the system’s application to the detection of LDFOs, along with an analysis of the detection thresholds, imaging optimization, and a discussion of the results in comparison to previous studies. Section 4 outlines the conclusions and potential future research directions.

## 2. Materials and Methods

### 2.1. Selection and Preparation of LDFOs and Infant Food Snacks Results

Six commercial infant products were selected according to their popularity. Table 1 lists the commercial infant products and LDFOs with their sizes. Puffed snacks, freeze-dried chips, and tofu waffles were obtained from Ildongfoodis Co. (Seoul, Republic of Korea). Rice snacks were acquired from Ssalgwajamaeul Co. (Gwangju, Republic of Korea). Sweet potato sticks were purchased from Firgi Co. (Pusan, Republic of Korea). Rice cereal was purchased from Maeil Dairies Co. (Seoul, Republic of Korea). Six LDFOs, namely, EPDM, PU, PVC, silicone, cockroach, and housefly were purchased from the local retail market (Daegu, Republic of Korea). They were selected as representative LDFOs based on reports from the Korea Ministry of Food and Drug Safety (MFDS) about the common contaminants found in domestic food products [25]. Indianmeal moths (Plodia interpunctella) were bred in a rearing chamber designed to mimic their natural environmental conditions at 25 °C and 75% RH [26].

### 2.2. Construction of the CW Sub-THz Imaging System and Experimental Setup

#### 2.2.1. Scheme of the CW Sub-THz Imaging System for LDFO Detection

**Figure 1:**
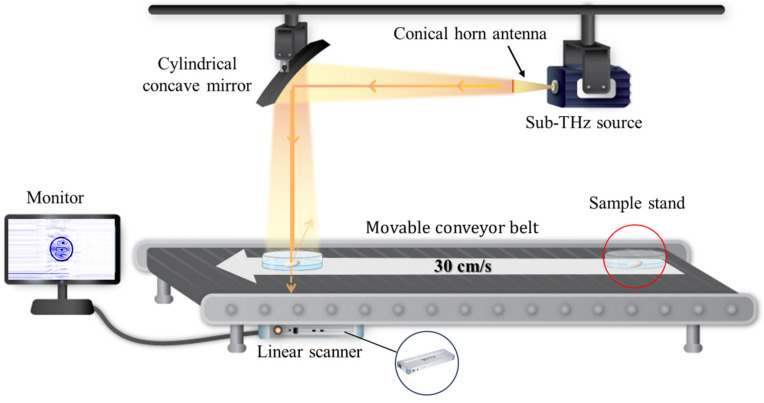
Diagram of the CW sub-THz imaging system.

Our research group constructed a CW sub-THz imaging system for the on-site detection of LDFOs in the food industry. As shown in Figure 1, the CW sub-THz imaging system comprises a sub-THz source, cylindrical concave mirror, linear scanner, movable conveyor belt, and monitor. The sub-terahertz source, cylindrical concave mirror, and linear scanner used in this system were manufactured by TeraFAST (TeraSense Group Inc., San Jose, CA, USA), and the components were systematically assembled and optimized to establish a customized system.

Once the sub-THz waves are emitted from the conical horn antenna at the source, the cylindrical concave mirror collects the emitted sub-THz waves and reflects them vertically to the linear scanner positioned underneath the movable conveyor belt at a speed of 30 cm/s. When the food with LDFOs arrives above the linear scanner, the reflected sub-THz waves pass through the food with LDFOs. The linear scanner, composed of 256 pixels arranged side-by-side lengthwise, has an excellent acquisition rate of up to 5000 lines per second, forming an image of food harboring LDFOs (Table 2). Depending on the composition, thickness, shape, and water content, the corresponding transmission images of food and LDFOs are displayed on a monitor.

#### 2.2.2. Optimization of Sub-THz Wave Imaging Through Attenuation and WBG Adjustments

The absorption, reflection, and transmission rates of sub-THz waves are influenced by chemical, physical, and structural properties such as the moisture content, density, and surface characteristics of food and LDFOs, necessitating experimental optimization for each food type [19]. For experimental optimization, the output power of the sub-THz wave was adjusted using attenuation values ranging from 0 to 9 dB to distinguish images of foreign objects within food. A set of samples containing three pieces of puffed snacks with different sizes of silicone placed underneath was prepared in a Petri dish. Subsequently, the Petri dish was placed on the sample stand, and the movable conveyor belt was operated at a speed of 30 cm/s. The transmitted sub-THz waves were scanned to obtain the images. After determining the attenuation values for each set of infant snacks, the brightness and contrast of each image displayed on the monitor were adjusted by controlling the White (W), Black (B), and Gamma (G) values within the range of 0–100 using TeraFAST software (TeraSense Group Inc., San Jose, CA, USA).

### 2.3. Detection of LDFO in Infant Snacks

Each set of infant snacks with a foreign object of 10 mm in size consisting of PU, PVC, EPDM, and silicone as well as cockroach, housefly, and Indianmeal moth were separately placed on a Petri dish. Each infant snack, except rice cereal, was placed over each LDFO. Rice cereals covered the LDFO with a single layer. The Petri dishes were placed on the movable conveyor belt, which operated at a speed of 30 cm/s. The transmitted sub-THz images of each sample were then scanned to evaluate the detectability of each LDFO.

### 2.4. Detection of Penicillium citrinum in Infant Snacks

Penicillium citrinum was provided by Dr. Lee’s laboratory, School of Life Science and Biotechnology, Kyungpook National University (Daegu, Republic of Korea). The strain was cultured in an incubator on potato dextrose agar (Difco Laboratories Co., Sparks, MD, USA) at 25 °C for 7 days to artificially contaminate the surface of various infant snacks, including puffed snacks and freeze-dried chips, using sterile swabs. Each infant snack inoculated with *P. citrinum* was then placed in an incubator at 25 °C for 4 days to allow the mold to cover approximately 30% of the total surface area and at 25 °C for 12 days to achieve 80% coverage. Finally, each infant snack contaminated with mold was placed on the movable conveyor belt operated at a speed of 30 cm/s, and the transmitted sub-THz images were scanned for comparison.

### 2.5. Determination of Detection Threshold

Three pieces of puffed snack and freeze-dried chip were separately arranged in a row on each Petri dish. PU, PVC, EPDM, and silicone of different sizes (2, 3, 4, 5, 7.5, and 10 mm) were placed underneath each infant snack. The Petri dishes were placed on a movable conveyor belt operated at a speed of 30 cm/s, and the transmitted sub-THz image was scanned to determine the threshold size of the LDFOs.

### 2.6. Determination of Detection Threshold in Stacked Infant Snacks

PU, PVC, EPDM, and silicone each sized at 3, 4, 5, 7.5, and 10 mm were placed beneath two layers of stacked puffed snacks and freeze-dried chips. The thickness was 8 mm for a single layer of freeze-dried chips and 16.1 mm for two layers; the puffed snack has a thickness of 10 mm for a single layer and 23.8 mm for two layers. The Petri dishes with stacked samples were positioned on a conveyor belt at 30 cm/s, and the transmitted sub-THz images were scanned to determine the threshold size of the LDFOs.

## 3. Results and Discussion

Our constructed CW sub-THz imaging system is suitable for on-site applications, especially with a compact form and excellent acquisition rate of up to 5000 lines per second (Figure 1 and Table 2). Additionally, the system operates within a nonionizing range of 0.1 THz with an output power of 800 mW, mitigating the radiation risks associated with X-ray detection and preserving the integrity of infant snacks using a non-destructive approach (Table 2).

**Figure 2:**
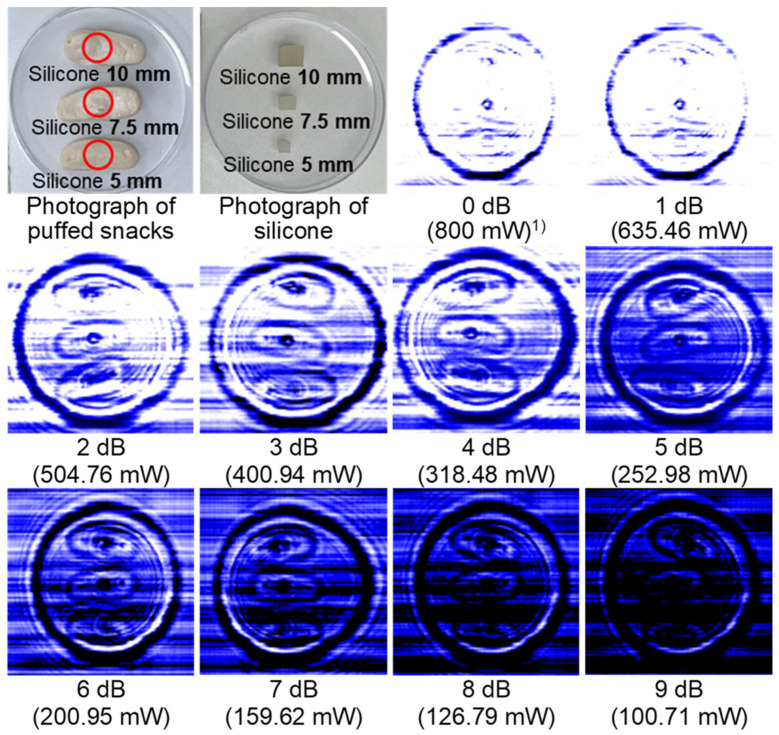
Transmission images of silicone placed underneath puffed snacks at various attenuation values. ^1)^ As the attenuation value increases, the output power of the sub-THz wave decreases.

The thickness, shape, and water content of food could influence the corresponding transmission image of both food and LDFOs [19]. Thus, the output power of the sub-THz wave was controlled by the attenuation value, ranging from 0 to 9 dB, for their distinct visualization. An increment in the attenuation value indicates a reduction in the output power of the sub-THz wave emitted by the source, which can be expressed as follows:

(1) $$y=10^{\frac{-x}{10}}\times 800$$

where *x* represents the attenuation value in dB and *y* represents the output power of the sub-THz wave (mW). A low attenuation value results in excessive penetration, causing the boundaries between the food and the LDFOs to become indistinct. Conversely, when the attenuation is too high, the transmitted wave does not have enough energy to adequately differentiate between the LDFOs and the food. As shown in Figure 2, at 0 and 1 dB, the penetration was too strong to distinguish between food and LDFOs. The attenuation values of 5–9 dB presented a difficulty in differentiating between silicone and puffed snacks owing to the relatively weak penetration of sub-THz wave. Thus, the optimum attenuation value of a puffed snack and freeze-dried chip with silicone was determined to be 3 dB; however, for other foods, it was determined to be 0 dB (Table 3). In addition, W, B, and G values were optimized and determined to enhance the visibility of LDFOs underneath various infant snacks (Table 3).

**Figure 3:**
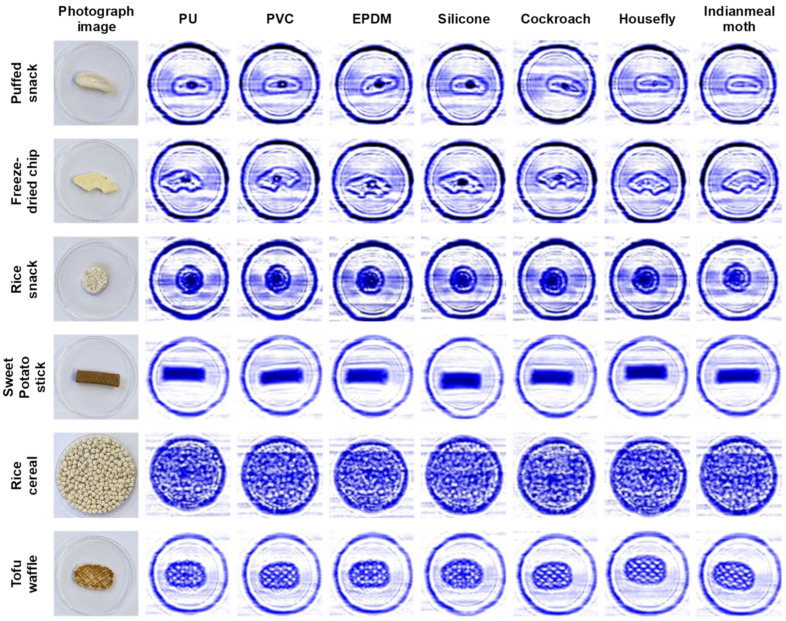
Transmission images of PU, PVC, EPDM, and silicone placed under each infant snack obtained using the CW sub-THz imaging system under optimized conditions.

**Figure 4:**
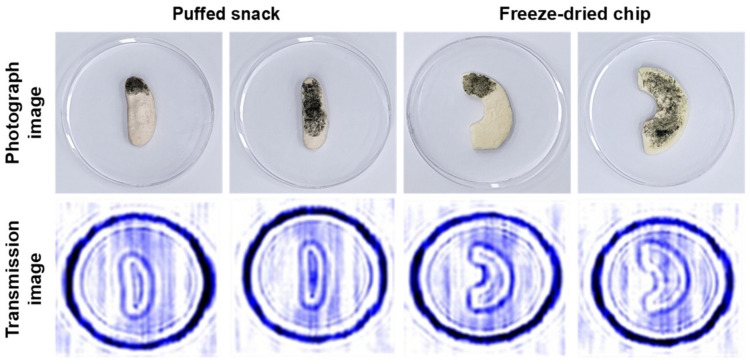
Transmission images of *P. citrinum* covering 30% and 80% of the surface of puffed snack and freeze-dried chip.

With the resulting optimized conditions for application, the transmission images of the seven LDFOs after their placement underneath each infant snack were obtained (Figure 3). As expected, all materials, PU, PVC, EPDM, silicone, cockroach, and housefly, except the Indianmeal moth, were detectable when placed underneath a puffed snack and freeze-dried chip. However, these LDFOs could not be detected in rice snacks, rice cereal, tofu waffles, or sweet potato sticks. Several food properties, such as irregular shape and thickness, material source, and composition, can also cause a scattering effect in both the transmission and reflection modes of a sub-THz wave [19]. The uneven surface of the tofu waffle and rice snacks probably impeded the even penetration of the sub-THz wave, thus hindering their obvious distinction from the infant snacks. In the case of rice cereal, the signal interference caused by the gaps between the snacks limited the differentiation between foreign objects and the infant snack. In addition, the dense surface of the sweet potato sticks presented a challenge for LDFOs, possibly due to the obstruction of sub-THz wave penetration. Therefore, the constructed CW sub-THz imaging system exhibited the potential to detect LDFOs in infant snacks with a low moisture content, an even surface, and a regular shape. Furthermore, the PU, PVC, silicone, EPDM, and the two types of insects (housefly and cockroach) appeared similar in shape, probably due to the relatively fast speed of conveyor belt movement and low sub-THz frequency. More precise differentiation between various LDFOs is possible by enhancing the frequency [12]. With respect to the detection of foreign objects in the food industry, the detection of their presence is more significant than the differentiation of LDFO types. Moreover, the detection of *P. citrinum* was unsuccessful at 30% or even 80% mold contamination (Figure 4). A failure to detect mold using the CW sub-THz imaging system may be attributed to the low density and very fine fiber-like structure of the mold.

**Figure 5:**
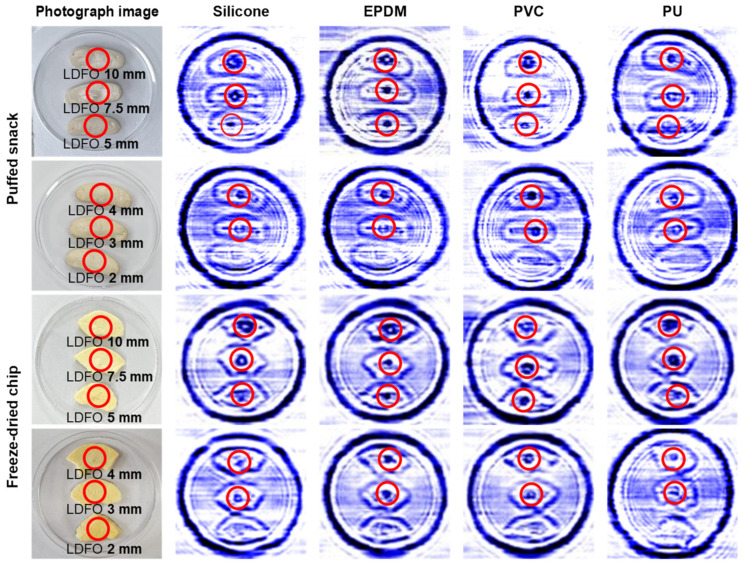
Transmission images of silicone, EPDM, PVC, and PU of various lengths. The red circles indicate the various lengths of silicone, EPDM, PVC, and PU placed underneath puffed snacks and freeze-dried chips.

Considering the result presented in Figure 3, puffed snacks and freeze-dried chips were selected for the determination of the detection threshold. The CW sub-THz imaging system successfully detected silicone, EPDM, PVC, and PU with a minimum size of up to 3 mm in both puffed snacks and freeze-dried chips (Figure 5), meeting the standard of 7 mm set by the United States Department of Agriculture (USDA) [27] for food products. However, LDFOs smaller than 3 mm in size were not clearly visible because of their high transmittance, which was attributed to the low thickness of these objects. This suggests that the sub-THz wave likely penetrated these smaller objects without adequate detection.

**Figure 6:**
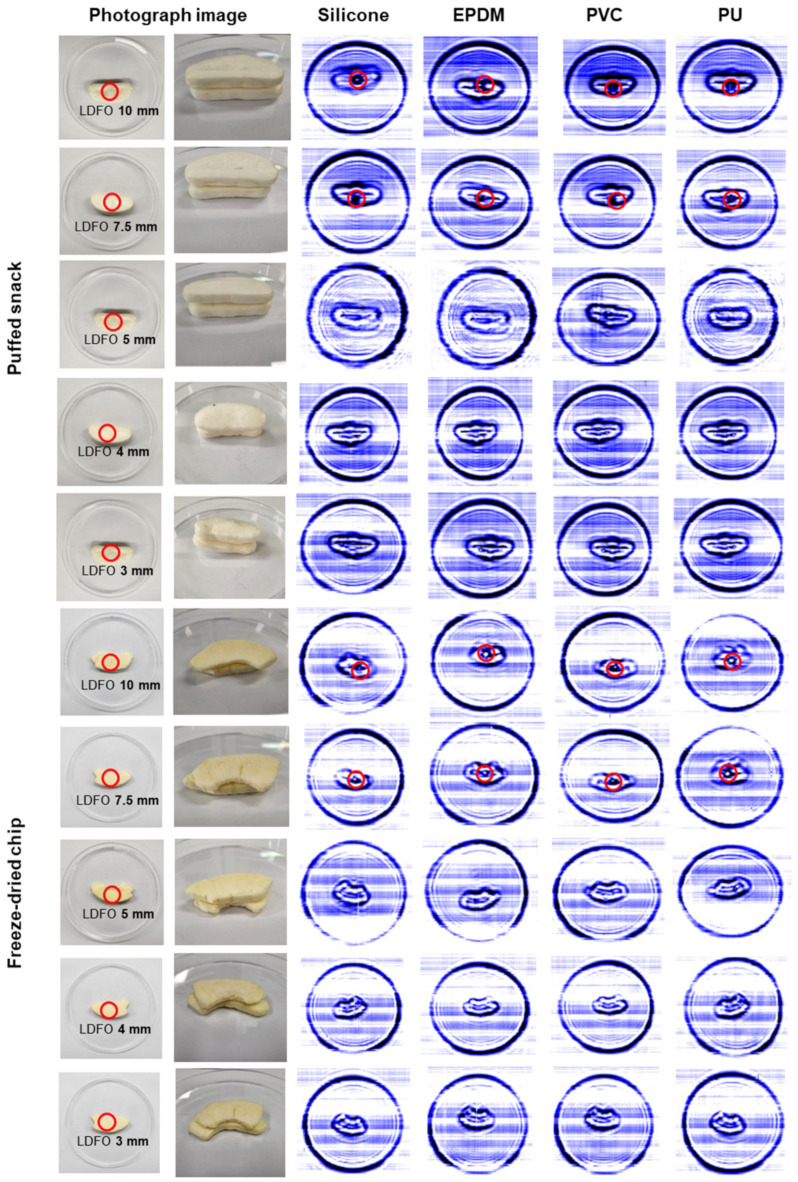
Transmission images of silicone, EPDM, PVC, and PU placed beneath two layers of puffed snacks and freeze-dried chips. The red circles indicate the placement of LDFOs underneath the stacked infant snacks.

The detection of LDFOs placed beneath two layers of stacked puffed snacks and freeze-dried chips was evaluated using a CW sub-THz imaging system. LDFOs of 7.5 mm and 10 mm in size were successfully detected in both snack types, with clear transmission images showing distinct outlines of PU, PVC, EPDM, and silicone (Figure 6). However, LDFOs of 5 mm in size were less clearly detected. The sub-THz wave appeared to have difficulty penetrating the smaller-sized foreign objects through the two layers of snacks, resulting in reduced contrast and less distinguishable images. This limitation in detecting smaller LDFOs can be attributed to the low transmittance of sub-THz waves through both the layered structure and the relatively smaller LDFOs. As the size of the LDFOs decreases, the transmittance of sub-THz waves through LDFOs increases, making it challenging to determine the presence of the LDFOs in the transmission images. These findings suggest that while the CW sub-THz imaging system is effective for detecting larger LDFOs in stacked food products, further optimization may be required to enhance the detectability of smaller objects, particularly in layered configurations. Future studies could focus on improving the system sensitivity for smaller LDFOs by exploring different wave frequencies or adjusting the imaging parameters for stacked food products.

In previous studies, dried food samples were used to avoid the influence of moisture content for THz detection. Ok et al. (2014) successfully detected maggot and cricket embedded in milk powder up to 8 and 35 mm in length, respectively, by using the CW THz system with a speed of 58 min/frame [23]. Sun et al. (2021) detected insects in finishing tea products using the TDS THz system, with an imaging time of 6 min/frame [1]. OK et al. (2019) detected mealworm, maggots, and polyethylene at lengths of 16, 9, and 5.5 mm, respectively, embedded in chocolate bars using a CW THz imaging system at a scanning speed of up to 30 min/frame [21]. Thus, our research presented the on-site detectability of various LDFO using a CW THz imaging system with a small size of 3 mm under a movable conveyor belt at a speed of 30 cm/s, which had faster detectability compared to several previous studies performed at a stationary stage [1,21,23]. Furthermore, our study confirmed the feasibility of using the CW sub-THz imaging system as a non-destructive, real-time, and on-site detection system for various LDFOs, particularly for infant snacks.

## 4. Conclusions

In this study, the CW sub-THz imaging system was constructed, optimized, and evaluated to detect LDFOs in infant snacks. The CW sub-THz imaging system has proven highly effective for detecting LDFOs in infant snacks and optimizing parameters such as attenuation and W, B, and G values to enhance visibility and differentiation. The system has successfully identified LDFOs as small as 3 mm in infant snacks with low moisture content, meeting USDA standards. In addition, the detectable size of the LDFOs was found to depend on the layer of infant snacks. Future studies on the detection of LDFOs in various packaging materials and at faster conveyor speeds are needed to evaluate the CW sub-THz imaging system for the practical use of the system. Furthermore, the integration of sub-THz technology with artificial intelligence-based image analysis is needed to enhance detection accuracy and enable the autonomous detection of LDFOs without human intervention. Specifically, the construction of an autonomous CW sub-THz imaging system by integrating the deep learning model will enhance the real-time and on-site detectability of LDFOs. This prospective construction promises to streamline processes, reduce error rates, and improve food safety, ensuring that our technology continues to meet evolving industry needs and maintains its position as an indispensable system for ensuring food safety.

## Figures and Tables

**Table 1:** Sizes of infant snacks and low-density foreign objects used in this study.

Materials	Products	Width (mm)	Length (mm)	Thickness (mm)
Food	Puffed snacks	25	61–66	10
	Freeze-dried chips	30	52–64	8
	Rice snacks	45	45	9
	Sweet potato sticks	24	70	14
	Rice cereal	7	7	7
	Tofu waffles	43	69	5
Low- density foreign objects	Ethylene–propylene–diene–monomer *	2–10 *	2–10	2–10
	Silicone *	2–10	2–10	2–10
	Polyvinyl chloride *	2–10	2–10	2–10
	Polyurethane *	2–10	2–10	2–10
	Housefly	4	8	4
	Indianmeal moth	5	5	2
	Cockroach	5	13	5

* EPDM, Silicone, PVC, and PU were divided into six distinct sizes, with dimensions ranging from 2 mm to 10 mm in width, length, and thickness (2 × 2 × 2 mm^3^, 3 × 3 × 3 mm^3^, 4 × 4 × 4 mm^3^, 5 × 5 × 5 mm^3^, 7.5 × 7.5 × 7.5 mm^3^, and 10 × 10 × 10 mm^3^).

**Table 2:** Constituent specifications of the CW sub-THz imaging system.

Equipment	Parameter	Specifications
**Sub-THz s** **ource**	Frequency	0.1 THz
	Power	800 mW
**Conical horn antenna**	Aperture size	25 × 6 mm^2^
**C** **ylindrical concave mirror**	Curvature radius	0.5 m
**Linear s** **canner**	Number of pixels	256
	Pixel size	1.5 × 3 mm^2^
	Imaging area	384 × 3 mm^2^
	Dimensions of device	450 × 160 × 44 mm^3^
	Image acquisition rate	5000 fps

**Table 3:** Determination of optimized values of attenuation, white, black, and gamma.

	Attenuation Value (dB)	White Value	Black Value	Gamma Value
Puffed snack	3	100	50	80
Freeze-dried chip	3	100	50	80
Rice snack	0	100	0	100
Sweet potato stick	0	100	0	100
Rice cereal	0	100	0	100
Tofu waffle	0	100	0	100

## Data Availability

Data are contained within the article.
